# Single vs. double intracoronary injection of mesenchymal stromal cell after acute myocardial infarction: the study protocol from a randomized clinical trial: BOOSTER-TAHA7 trial

**DOI:** 10.1186/s13063-022-06276-y

**Published:** 2022-04-12

**Authors:** Armin Attar, Fatemeh Nouri, Arash Yazdanshenas, Kamran Hessami, Massoud Vosough, Alireza Abdi-Ardekani, Peyman Izadpanah, Mani Ramzi, Javad Kojouri, Gholamreza Pouladfar, Ahmad Monabati

**Affiliations:** 1 Department of Cardiovascular Medicine, TAHA clinical trial group, Shiraz University of Medical Sciences, Shiraz, 71344-1864 Iran; 2 Student Research Committee, Shiraz University of Medical Sciences, Shiraz, Iran; 3 Clinical Microbiology Research Center, Nemazee Hospital, Shiraz University of Medical Sciences, Shiraz, Iran; 4 Department of Regenerative Medicine, Cell Science Research Center, Royan Institute for Stem Cell Biology and Technology, ACECR, Tehran, Iran; 5 Department of Cardiovascular Medicine, Shiraz University of Medical Sciences, Shiraz, Iran; 6 Hematology Research Center, Shiraz University of Medical Sciences, Shiraz, Iran; 7 Department of Pathology, Shiraz University of Medical Sciences, Shiraz, Iran

**Keywords:** Regenerative Medicine, Cell therapy, Myocardial infarction, Mesenchymal stromal cell, Intracoronary injection, Acute myocardial infarction

## Abstract

**Background:**

Meta-analysis from previous studies have shown that treatment with mesenchymal stromal cell (MCSs) may increase the left ventricular ejection fraction (LVEF) after acute myocardial infarction (AMI) by 3.84%, and the effect is greater in those who are not aged and have developed a reduced LVEF. However, it seems that MSC transplantation does its effect through an indirect paracrine effect, and direct differentiation to the cardiomyocytes does not occur. Therefore, it can be hypothesized that this paracrine effect would be augmented if repeated doses of MSC are transplanted. This study is conducted to compare single vs. double injection of MSCs.

**Methods:**

This is a single-blind, randomized, multicenter trial aiming to determine whether intracoronary infusion of double doses of umbilical cord-derived Wharton’s jelly MSCs (WJ-MSCs) improves LVEF more after AMI compared to single administration. Sixty patients 3 to 7 days after AMI will be enrolled. The patients should be under 65 years old and have a severe impairment in LV function (LVEF < 40%). They will be randomized to three arms receiving single or double doses of intracoronary infusion of WJ-MSCs or placebo. The primary endpoint of this study is assessment of improvement in LVEF at 6-month post intervention as compared to the baseline.

**Discussion:**

This investigation will help to determine whether infusion of booster (second) dose of intracoronary WJ-MSCs in patients with AMI will contribute to increasing its effect on the improvement of myocardial function.

**Trial registration:**

Iranian Registry of Clinical Trials (www.IRCT.ir) IRCT20201116049408N1. Registered on November 26 2020

## Background

Myocardial infarction (MI), a common presentation of coronary artery disease, is the main cause of death in the developed countries [1]. Over the past few decades, a rise in the incidence of heart failure (HF) was observed in contrast to the reduction in the mortality rate after MI [2]. Occurrence of HF in hospitalized patients for an acute myocardial infarction (AMI) ranged from 14 to 36% in previous studies [3]. HF has an important impact on the healthcare system. HF accounts for 6 million patients, 300,000 death cases, and about $40 billion healthcare costs in the USA per year [4].

In spite of the current guideline-directed therapy [5], mortality and morbidity of post-MI heart failure is quite high [6, 7]. Although current managements for HF are prolonging the patients’ life while improving their symptoms, they do not restore the normal histologic architecture and induce regeneration in the damaged cells. Therefore, improving confirmed treatments and developing further approaches to treat patients with post-MI heart failure are strongly required [8]. One approach has pointed at stem cell-based therapies [9]. Cell therapy provides a potential approach to fundamentally reconstructing the dead myocardial cells.

### Cell-based therapy in cardiovascular disease

Cell-based therapy in cardiovascular diseases was initiated in the late 1990s when preclinical studies revealed the possibility of transplanting the skeletal myoblasts [10] and fetal cardiomyocytes [11] into ischemic myocardium. Afterwards, the bone marrow (BM) cell implant in murine models of MI was reported [12, 13]. Cell therapy moved forward to human studies with outstanding speed, using skeletal myoblasts in patients with HF in 2001 [14] and BM-derived cell transplantation in acute MI in 2002 [15]. Since then, many studies on animals and humans have been performed to assess different cell types and their ability to repair cardiac and vascular damage in the settings of MI, cardiomyopathy, etc.

### Mesenchymal stromal cells (MSCs)

MSCs are a population of cells initially isolated from the BM and have been found in other organs and tissues such as the heart, Wharton’s jelly, and adipose tissue [16]. Due to the availability of these resources, these tissues are becoming the dominant source for isolation of MSC for clinical uses [17]. Furthermore, the safety of MSCs-therapy from these origins has been confirmed previously [18]. Because of their desirable features, such as simplicity of isolation and ex vivo growth, in vitro stemness characteristics and an immune privileged feature, MSCs, are progressively used in clinical trials of stem cell therapy [19]. In the POSEIDON trial, it was shown that transplantation of allogenic as compared to autologous MSCs is completely safe and is equally effective [20]. In the TAC-HFT trial, it was shown that MSC are about twice as much effective as the bone marrow-derived mononuclear cells (BM-MNCs) [21]. Based on these findings, it seems that MSCs are the most readily available yet and efficient cells for regenerative therapeutic approaches in cardiology.

### Cell therapy in acute myocardial infarction

Most of the studies on cell therapy in AMI are done by BM-MNCs. Findings from those trials have cleared the path through other resources and answered many questions. Based on TIME trials, it was noticed that the best transplantation time after AMI is within 3 to 7 days just after the attack [22, 23]. Based on the meta-analysis by Fisher and colleagues, treatment with BM-MNC would increase left ventricular ejection fraction (LVEF) after AMI by 2.72%. It is of high importance to point that in the selected population of patients aged under 55 and LVEF under 37%, this treatment yielded survival and functional benefits as well [24].

Clinical trials using MSCs in AMI are controversial but encouraging. The largest clinical trial conducted in the field was done by Gao and coworkers on 116 patients. They found that umbilical cord Wharton’s jelly-derived MSCs could enhance the LVEF by nearly 5% [25]. A meta-analysis in the field showed similar results of improving LVEF by 3.84% [26]. These findings are parallel to the results of TAC-HFT trial that showed MSCs were nearly twice as much effective as BM-MNCs [21].

### Mechanism of action of cell therapy

The exact mechanisms accounting for the beneficial effects of using stem cells in HF in preclinical and clinical studies are not clear. The data supporting the theory on differentiation of the transplanted cells as a mechanism of improvement in the recipient heart are very poor; even if all of the remaining cells are transformed into the cardiomyocytes, it would not be sufficient to account for the useful effects reported [27]. Differentiation of the transplanted cells into new vessels has been observed in different cells, such as MSCs [28], and it has been suggested that vasculogenesis may result in rescuing the cardiomyocytes in the hypoxic area. It is challenging to imagine (consider) how the vasculogenesis mechanism could be a main mechanism in patients who already had successful coronary revascularization after an AMI; however, it is obvious that this phenomenon can be responsible for some of the advantageous effects of cell therapy. Recently, the paradigm has shifted from these mechanisms to the paracrine effect theory, which suggests that most of the beneficial effects after cell therapy are obtained through signals such as cytokines that are released in a paracrine signaling by the injected cells and alter the nearby cells and the recipient heart [29].

### Hypothesis generation

If the beneficial effects of MSC therapy could be explained by paracrine effect, increasing the frequency of cell injections would be associated with more useful effects on the recipient heart.

### Study design

**Fig. 1:**
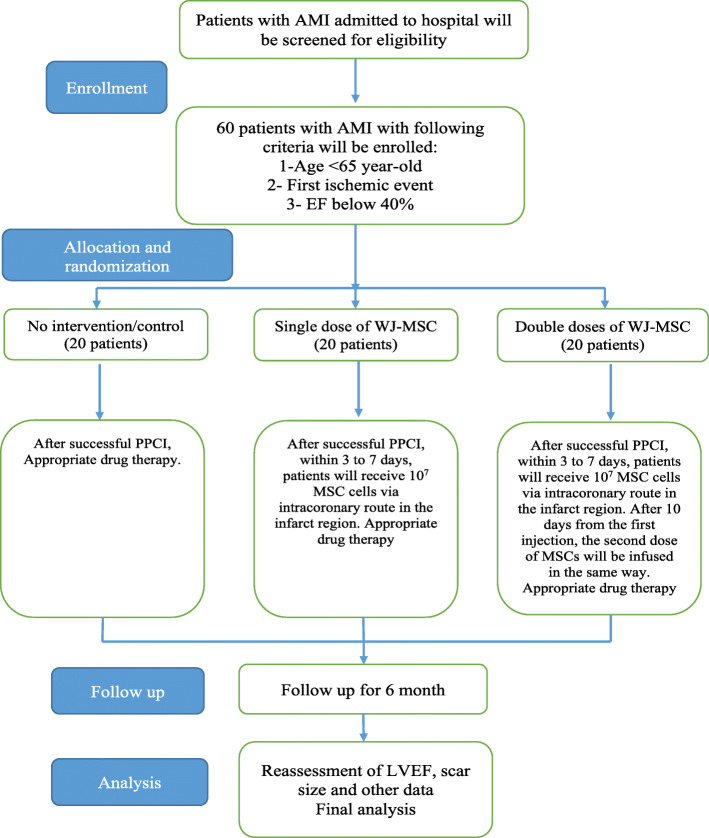
SPIRIT flow diagram of study

This is a randomized, single-blind, multicenter phase II trial which aimed to determine whether the intracoronary infusion of double doses of the umbilical cord Wharton’s jelly tissue-derived MSCs as compared to one or no injection demonstrates greater effect on LVEF after an AMI when administered additionally to the standard management. This study has been approved by the “Ethics Committee of Shiraz University of Medical Sciences” (the code: IR.SUMS.REC.1399.406) and registered in the Iranian Registry of Clinical Trials website (https://www.irct.ir/) by the code IRCT20201116049408N1. The study protocol will be reported based on Standard Protocol Items: Recommendations for Interventional Trials (SPIRIT) guidelines (Online supplement 1). Figure 1 shows SPIRIT flow diagram of study.

### Sample size determination

According to the objectives and type of the study and citing previous studies in this field, taking into account the 5% error, 90% power, 3% difference between placebo group and single dose injection group, and another 3% difference between single and double injection groups after the 6 month follow-up period and 1.3 standard deviation and 1:1:1 ratio, the sample size will be estimated in each group using the formula $$ n=\frac{2{s}^2{\left({z}_{1-\raisebox{1ex}{$\alpha $}\!\left/ \!\raisebox{-1ex}{$2$}\right.}+{z}_{1-\beta}\right)}^2}{{\left(\partial \right)}^2} $$ . Due to the length of the study and repeated measurements, using the formula $$ {n}^{\prime }=n\times \frac{1}{1-p} $$, and a drop of 15%, the sample size is decided 16 in each group. Finally, considering that in this study, three groups will be compared and according to the formula, $$ {n}^{\prime }=n\times \sqrt{k} $$, 20 subjects will be needed in each group, so a total of 60 patients will be selected. In the above formula, the values of *z* are constant and equal to 97.5th percentile and 80th percentile of the standard distribution. The ratio of people in the two groups and the effect size are the value divided by the standard deviation.

### Study participants

Sixty patients will be enrolled in this trial; they should have been hospitalized due to anterior ST elevation myocardial infarction (STEMI), undergone successful primary percutaneous coronary intervention (PCI), and had a LVEF < 40%. Centers participating in the trial are the Alzahra Heart Hospital (Shiraz, Iran), Nemazee Hospital (Shiraz, Iran), and Shahid Faghihi Hospital (Shiraz, Iran). Inclusion and exclusion criteria are listed below:

Inclusion criteria:

1. Ages 20–60 years
2. Genders: Both
3. First MI within 3 to 7 days
4. Post-AMI LVEF less than 40% as assessed by echocardiography
5. Negative pregnancy test (in women with childbearing potential)
6. Written informed consent

Exclusion criteria:

1. History of prior anterior myocardial infarction
2. Patients with regional wall motion abnormalities in the non-infarct region
3. Prior coronary artery bypass graft (CABG) surgery
4. Patients with significant valve disease, defined as stenosis or regurgitation graded as greater than moderate (2+)
5. Patients with another etiology of LV dysfunction (known/suspected non ischemic cardiomyopathy, previous anthracycline therapy, known ethanol abuse (greater than 6 oz. ethanol/day on a regular basis)
6. Poor echocardiography window
7. Active infection or history of recurrent infection or positive test for syphilis (RPR), hepatitis B and C (HBsAg, Anti-HCV), HIV, and HTLV-1
8. Documental terminal illness or malignancy
9. Previous bone marrow transplant
10. Autoimmune disease (e.g., lupus, multiple sclerosis)

### Randomization and study blinding

Patients will be randomized 1:1:1 to receive WJ-MSCs using permuted block randomization with block size 6. The outcome assessors will be blinded to the assignment (single-blind). The randomization process will be done by using a web-based randomization service.

### Intervention

The cGMP certified clinical-grad hWJ-MSCs produced by Cell Tech Pharmed Co. Ltd. (Tehran, Iran) are transported to the ward on the infusion day. The hWJ-MSCs were suspended in 0.9% normal saline. A qualified person will issue the certificate of analysis for each batch. The release of cells is in compliance with product specifications. The shipment will be handled considering validated standard operation protocols.

Forty patients in the two intervention arms will receive one intracoronary dose of 10^7^ WJ-MSCs infusion, and the control group will be administered only the conventional supportive treatment. For 20 patients among those who had received the first dose, the second dose of 10^7^ WJ-MSCs infusion was done within 10 days after the first injection.

A total of 60 patients in the three groups will be randomized in a 1:1:1 pattern to receive two doses of WJ-MSCs (*n* = 20; 2 × 10^7^ cells) or one dose of WJ-MSCs (*n* = 20; 1 × 10^7^ cells) in combination with conventional supportive treatments or conventional supportive treatments alone (*n* = 20).

When the cells are ready for infusion, the patients will return to the cardiac catheterization laboratory. The total number of 10^7^ WJ-MSCs will be infused via intracoronary route. If the activated clotting time is under 200, a weight-based bolus dose of heparin will be used.

Therapeutic 6 Fr guiding catheter will be inserted into the left main artery. Two hundred micrograms of nitroglycerin will be infused via the guiding catheter. Angiography of LAD will be done, and TIMI flow will be documented. 0.014-inch soft-tipped guidewire wire will be inserted into LAD at distal edge of the stent. An over-the-wire balloon will be passed and placed within the stented area. It will be inflated to occlude the vessel. The guiding wire will be removed from the microinfusion device. Infusion syringe will be connected to the infusion catheter, and infusion of MSCs will be initiated at 2.5 ml/min. Occlusion with balloon catheter will be done using low pressure inflation (2–4 bar). Before each cell infusion, total occlusion of the coronary artery will be confirmed by injection of dye in the selected vessel. When one third volume of the cells is infused, the infusion pump will be ceased, and the contrast agent is injected into the guiding catheter to assess the TIMI coronary flow. This will be done again for the second one third volume. When cell infusion is completed, the coronary flow wire will be placed via the microinfusion catheter. After 10 days, this procedure will be repeated for patients in the third group.

### Patient follow-up and study endpoints

During the hospital course, patients will be visited every day by a cardiologist. Performed physical examinations and vital signs will be recorded. We will monitor the patients during admission and any signs of arrhythmia, pulmonary emboli, and coronary artery injury will be evaluated and recorded. Blood samples are drawn for measurement of fasting blood sugar (FBS), complete cell blood count (CBC), urea and electrolytes, liver function tests (LFTs), creatine kinase, and cardiac troponin T- and C-reactive protein. An electrocardiogram (ECG) test is also taken. Before the first cell infusion, cardiac magnetic resonance (CMR) imaging and echocardiography will be done. After cell infusion, patients will be discharged from the hospital with a prescription for beta-blocker, angiotensin-converting enzyme (ACE) inhibitor, aldosterone antagonist, aspirin, ticagrelor, statin, and nitrate spray or tablets to take as required and cardiac rehabilitation program. Blood tests, an ECG, and physical exams are again done at the 10th day (at time of admission for second cell infusion), 3-month, and 6-month visits. At the last visit, echocardiography and CMR will be re-performed. All the tests will be recorded.

The improvement in LVEF after 6 months will be the primary efficacy endpoint for this study. Secondary endpoints will be the infarct size of the infarcted and salvaged myocardium at 6 months as assessed by CMR, changes in LV function, left ventricular mass (LVM) index, left ventricular end diastolic volume (LVEDV), left ventricular end systolic volume (LVESD), and global longitudinal strain (GLS) at 6 months as determined by echocardiography.

### Cardiac MRI

CMR will be performed for each patient 3 days and 6 months after primary PCI. For evaluation of the ventricular function and volume of cine-CMR, and for determining the microvascular obstruction and size of the infarcted myocardium evaluation, delayed enhancement (DE)-CMR will be used. T2 imaging will be used to assess the myocardial salvage and infarct size. Optimization of myocardial nulling will be done by the inversion time. Images will be evaluated in a blinded way by an expert operator. Scar and edema volumes will be determined by manually tracing the endocardial and epicardial contours after semi-automated selection of the normal remote myocardium per slice.

### Echocardiography

For evaluation of LV systolic function, echocardiography will be performed. By using wall motion score and Simpson’s rule, EF will be calculated. LV function will be evaluated on the day of hospitalization before cell infusion and 6 months after cell infusion to determine the changes in EF. GLS will be measured using automated formulas in standard views.

### Adjudication of potential endpoints

All measurements will be evaluated and judged by an independent, blinded expert. In the case of poor recordings, the expert chooses whether the measurement has adequate quality to be used for endpoint assessment or not. Measurements of inadequate quality will not be used in the analysis as missing measurements. Potential major adverse cardiac events (MACE) will be evaluated by an independent, blinded safety committee. All laboratory data and adjudications will be finalized and moved into the computer database prior to unblinding study.

### Statistical analysis

All data will remain anonymous before the analysis, which will be done by an independent, expert member of the department not involved in the trial. Based on the findings from previous meta-analysis, a 3% improvement in ejection fraction, as measured by CMR after 6 months, will be considered significant [26]. An intention-to-treat analysis will be performed. We will also analyze the baseline demographic and clinical characteristics for each arm of the study. Mean and standard deviation will be used for continuous variables, and categorical data will be shown by frequencies and percentages. One-way analysis of variance (ANOVA) will be used to perform comparisons of LVEF changes after 6 month (primary endpoint) and also other outcomes between the treatment arms regarding the followed by post hoc Tukey’s test for comparisons of the double-doses-infusion arm with single-dose-infusion group and placebo control-treated groups individually. Only cases with complete follow-up will enter the final analysis. Paired *t*-test will be performed for within-group comparisons. Estimated treatment effect will be reported with 95% CI. All *p* values will be two-sided. Safety events such as the incidence of MACE (death, recurrent AMI, ICD insertion, non-target vessel revascularization…) and serious adverse events (SAE) will be compared among the three arms, and Kaplan-Meier curves will be used to show the pattern of events during the 6-month follow-up. Using Cox’s proportional hazards model, statistical significance and 95% CIs will be reported.

### Adverse events reporting

The Executive Committee of the Study will report the case to an independent Data and Safety and Monitoring Board (DSMB) for monitoring the patient safety. The DSMB can suggest that the trial should be ceased early due to concerns about the patients’ safety or because the major research question has been answered. The DSMB will monitor the safety events all over the trial which will include unexpected SAEs, mortality, liposuction complications, intracoronary infusion complications, and serious or life-threatening arrhythmia. Every 3 months, DSMB statistician will report the patient safety events by tabulations for treatment groups. Mortalities are reported for every individual.

### Ethical considerations

All ethical considerations of this trial were discussed and approved in institutional review board of Shiraz University of Medical Sciences (IR.SUMS.REC.1399.406). Taking informed consent in the acute setting is an important ethical issue with this type of trial. To deal with this issue, we will obtain the consent forms when the patients are in a stable clinical condition and had sufficient time to recover from sedation or analgesics. Using the patients’ umbilical cord Wharton’s jelly tissue-derived MSCs will have the risk of an allergic reaction near zero. Furthermore, using a low balloon inflation pressure and a divided infusion time prevented intracoronary cell infusion complications.

## Discussion

Currently, all the therapeutic measures after AMI are focused to prevent remodeling and further myocyte loss [5]. Regenerative medicine and cell therapy have provided new hopes to not only prevent the remodeling process but also reversing the process by providing new functional cells and increasing the cardiac function capacity [25].

The results from preliminary studies were not very encouraging, and the potential of stem cells for cardiac regeneration became doubted [9]. However, further meta-analyses showed that in certain populations this therapy may be effective. In a meta-analysis from the Cochrane database, it was shown that treatment of young AMI patients with a reduced LVEF with BM-MNCs not only is effective in increasing LVEF but also may have survival and functional benefits [24].

Studies of MSCs have shown greater promise in the field as compared to BM-MNCs. In the TAC-HFT trial, it was shown that MSC are about twice as much effective as the bone marrow-derived mononuclear cells (BM-MNCs) [21]. Meta-analysis of MSC trials showed similar results and depicted a improving LVEF by 3.84% [26] as compared to 2.72% effect seen by BM-MNCs [24].

There is still debate regarding the mechanism by which stem cells, particularly MSCs, impose their therapeutic effects. Current paradigm suggests the paracrine effect theory indicating that most of the beneficial effects after cell therapy are obtained through signals such as cytokines that are released in a paracrine signaling by the injected cells and alter the nearby cells and the recipient heart [29].

Our trial by including the selected patients who are young and have developed with a reduced LVEF after AMI provides important insights in the field in two ways. First, it will show us whether the findings from meta-analysis that indicated cell therapy effect is confined to special population of patients post AMI are reproducible in a clinical trial or not. Second, by performing the second dose injection, it provides us with clues regarding the mechanisms of how MSC may help in the regeneration of cardiac tissue.

## Dissemination

The study will adhere to the principles outlined in the *Declaration of Helsinki*. Data monitoring will be finished by November 2021. Primary and secondary analysis will be initiated after data collection is completed. The results will be ready for submission in December 2021. The manuscript of the trial will be submitted due to the CONSORT statement. According to recommendations, this trial has been registered with a public registry IRCT.ir.

## Trial status

This trial is still recruiting the patients. Recruitment started on January 2021, and enrollment is estimated to end by November 2021. This protocol is version 2 approved on October 2020. The COVID-19 pandemics did not influence the patient enrollment in this trial.

## Data Availability

After publication of the final results, the data will be available based on the requests.

## Abbreviations

MCSs: Mesenchymal stromal cell
LVEF: Left ventricular ejection fraction
AMI: Acute myocardial infarction
WJ-MSCs: Wharton’s jelly mesenchymal stromal cell
MI: Myocardial infarction
BM: Bone marrow
HF: Heart failure
BM-MNCs: Bone marrow-derived mononuclear cells
EF: Ejection fraction
CMR: Cardiac magnetic resonance
GLS: Global longitudinal strain
DE: Delayed enhancement
MACE: Major adverse cardiac events
SAE: Serious adverse events
DSMB: Data and Safety and Monitoring Board
